# Novel foetal echocardiographic image processing software (5D Heart) improves the display of key diagnostic elements in foetal echocardiography

**DOI:** 10.1186/s12880-020-00429-8

**Published:** 2020-04-03

**Authors:** Wan-Yu Hu, Jin-Hong Zhou, Xiao-Ying Tao, Shi-Yan Li, Bei Wang, Bo-Wen Zhao

**Affiliations:** 1 Department of Diagnostic Ultrasound & Echocardiography, Sir Run Run Shaw Hospital, Zhejiang University School of Medicine, No. 3 East Qingchun Road, Hangzhou, 310016 People’s Republic of China; 2 Department of Ultrasonography, Quzhou People’s Hospital, Quzhou, People’s Republic of China; 3 Department of Ultrasonography, Jinhua Municipal Central Hospital, Jinhua, People’s Republic of China

**Keywords:** 5D Heart, Foetal intelligent navigation echocardiography, Diagnostic element, Foetal echocardiography, Congenital heart disease

## Abstract

**Background:**

To evaluate the clinical value of foetal intelligent navigation echocardiography (5D Heart) for the display of key diagnostic elements in basic sections.

**Methods:**

3D volume datasets of 182 normal singleton foetuses were acquired with a four chamber view by using a volume probe. After processing the datasets by using 5D Heart, eight cardiac diagnostic planes were demonstrated, and the image qualities of the key diagnostic elements were graded by 3 doctors with different experiences in performing foetal echocardiography.

**Results:**

A total of 231 volume datasets acquired from the 182 normal foetuses were used for 5D Heart analysis and display. The success rate of 8 standard diagnostic views was 88.2%, and the success rate of each diagnostic view was 55.8–99.2% and 70.7–99.0% for the random four chamber view as the initial section and for the apical four chamber view as the initial section, respectively. The success rate of each diagnostic element in the 8 diagnostic sections obtained by 5D Heart was 58.9%~ 100%. Excellent agreement was found between experienced sonographers and less-experienced sonographers (kappa> 0.769). Inter- and intra-observer agreement were substantial to near-perfect, kappa values ranging from 0.612 to 1.000 (Cohen’s kappa).

**Conclusions:**

5D Heart can significantly improve the image quality of key diagnostic elements in foetal echocardiography with low operator dependency and good reproducibility.

## Background

Congenital heart diseases (CHDs) are the leading cause of organ-specific birth defects, with a prevalence of 3–8 per 1000 live births, and they are also the leading cause of infant mortality due to congenital malformations [1–4]. Indeed, despite almost universal access to sonographic screening during pregnancy, only 28% of major CHDs are detected prenatally [1, 3, 5]. Some of this variation can be attributed to differences in examiners’ experiences, maternal obesity, transducer frequency, abdominal scars, gestational age, amniotic fluid volume and foetal position [6–8]. The difficulties of prenatal diagnosis are generally attributed to the complex anatomy of the heart as well as its motion and small size. Adequate examination of the foetal heart, depending on the foetal position, is time-consuming and expertise-dependent [3, 6, 9–11]. An increasing number of methods and technologies have emerged to improve the diagnosis of foetal heart diseases. Recently, increasing evidence has suggested that three-dimensional (3D) sonography [12, 13] and four-dimensional (4D) sonography with spatiotemporal image correlation (STIC) facilitate examination of the foetal heart [13–16] and can also be used to evaluate foetal cardiac function and CHDs [12, 17, 18].

STIC is a new approach for the clinical assessment of the foetal heart [4, 10, 12, 19]. It offers an easy-to-use technique to acquire data from the foetal heart and to aid in visualization with both two-dimensional (2D) and 3D cine sequences. Previous research has validated a volume dataset of a foetus within 7.5–15 s with STIC [4, 18, 19]. After the original datasets of the foetal heart are acquired, they can be analysed online or offline. However, extracting and displaying the recommended diagnostic planes from a volume dataset that can be dissected in many ways (i.e., planes and foetal position) requires in-depth knowledge of foetal anatomy and is truly difficult, operator-dependent, and time-consuming [12, 17, 18]. Moreover, planes and cardiac structures may be difficult to recognize when the anatomy is abnormal or when the foetus is in a transverse position. Special training is required in order to successfully complete the 3D image display and analysis [3, 20].

As conventional 2D echocardiography visualizes only the length and width of the heart, 3D images were based on a series of 2D images, showing a visual heart through three orthogonal planes. 4D techniques use the added dimension of time to present a real-time 3D image. 5D Heart uses intelligent 4D images. 5D Heart is the latest STIC technology based on foetal intelligent navigation echocardiography (FINE) technology [21–23]. It can obtain volume datasets systematically and efficiently assist users in displaying cardiac diagnostic planes [22]. The current version of the FINE method is able to generate eight foetal echocardiography views automatically [21, 22]. However, its methodology and diagnostic value, to the best of our knowledge, have not been fully investigated. In this study, we aimed to explore the use of 5D Heart in displaying diagnostic elements in 8 diagnostic sections and to evaluate its clinical value in prenatal ultrasound screening for CHDs to provide methodological guidance in terms of clinical applications.

## Methods

### Study population

A total of 209 foetuses were randomly selected from patients with singleton pregnancies in the second and third trimesters who had sonographic examinations in our outpatient clinic (Sir Run Run Shaw Hospital, Zhejiang University College of Medicine) from January 2017 to March 2017. Among them, satisfactory volume datasets could not be obtained from 27 foetuses due to maternal obesity, foetal motion and other factors. Therefore, the number of selected cases was 182, and the number of effective volume datasets was 231. The gestational ages ranged from 17 to 38 weeks (mean, 27.59 ± 3.80 weeks), and the pregnant women’s ages were between 16 and 41 years old (mean, 28.86 ± 4.42 years). In this study, all pregnant women had an accurate menstrual history, or the sonographic age was consistent with the gestational age calculated by the menstrual history (less than 2 weeks). Biparietal diameter and femoral diaphysis length were used to estimate the gestational age. If there was a difference of more than 1 week, the sonographic age was used [24, 25]. None of the foetuses included in this study had any cardiac or noncardiac abnormalities, including chromosomal abnormalities, according to a third-level 2D echocardiographic examination, and gestational age estimated by ultrasound did not match gestational age estimated by menstrual history (≥2 weeks). Foetuses with thick nuchal translucency at 11~13 weeks + 6 days and mothers with gestational anaemia, diabetes, hypertension and other diseases were excluded regardless of the pregnancy outcome or postnatal follow-up findings. This study was conducted at Sir Run Run Shaw Hospital, with approval from the institute’s ethics committee. All participants gave informed written consent.

### Acquisition of 3D volumes

The 3D volume datasets of the foetal hearts were obtained by an expert (B.W.Z., Observer C) via sonography and foetal echocardiography using a curved array transducer (1–8 MHz, WS80A Expert series, Samsung Medical Systems, Korea). First, routine screening with 2D ultrasound of the foetal heart was performed to exclude foetal cardiac structural abnormalities, and the cardiac rate and regular rhythm were confirmed. Second, an attempt was made to obtain clear 2D images via foetal echocardiography. The frame rate was maximized by decreasing the depth, narrowing the sector width, and placing a single focal zone at the level of the foetal heart. The normal rate ranged from 120 to 160 beats per minute. 3D volume datasets of the foetal heart were acquired from a transverse apical four chamber view of the foetal chest. Images were magnified until the heart filled at least one-third to one-half of the displayed screen. Finally, participants were asked to momentarily hold their breath during 5D Heart acquisition, and volume datasets were acquired by automatic scanning. Acquisition times ranged from 7 to 10 s depending on the size of the foetal heart and its distance from the probe, while the angle of acquisition ranged between 35° and 45° depending on the gestational age. The imaging volume was set to include the spine, a complete four chamber view, the abdominal aorta, the upper mediastinum and the gastric bubble but to exclude other unnecessary foetal or maternal body parts [20, 22, 23]. If the foetal position changed, another volume dataset could be acquired and taken into account in a different volume dataset. Therefore, more than one volume dataset was acquired from one foetus. After the acquisition, the volume datasets were stored on a hard drive in preparation for processing and analysing. The data were processed by a physician (J.H.Z., Observer A) with less experience but who had been engaged in ultrasound diagnosis for more than 5 years and had at least six months of systematic foetal echocardiography training.

### Testing with the FINE method

**Fig. 1:**
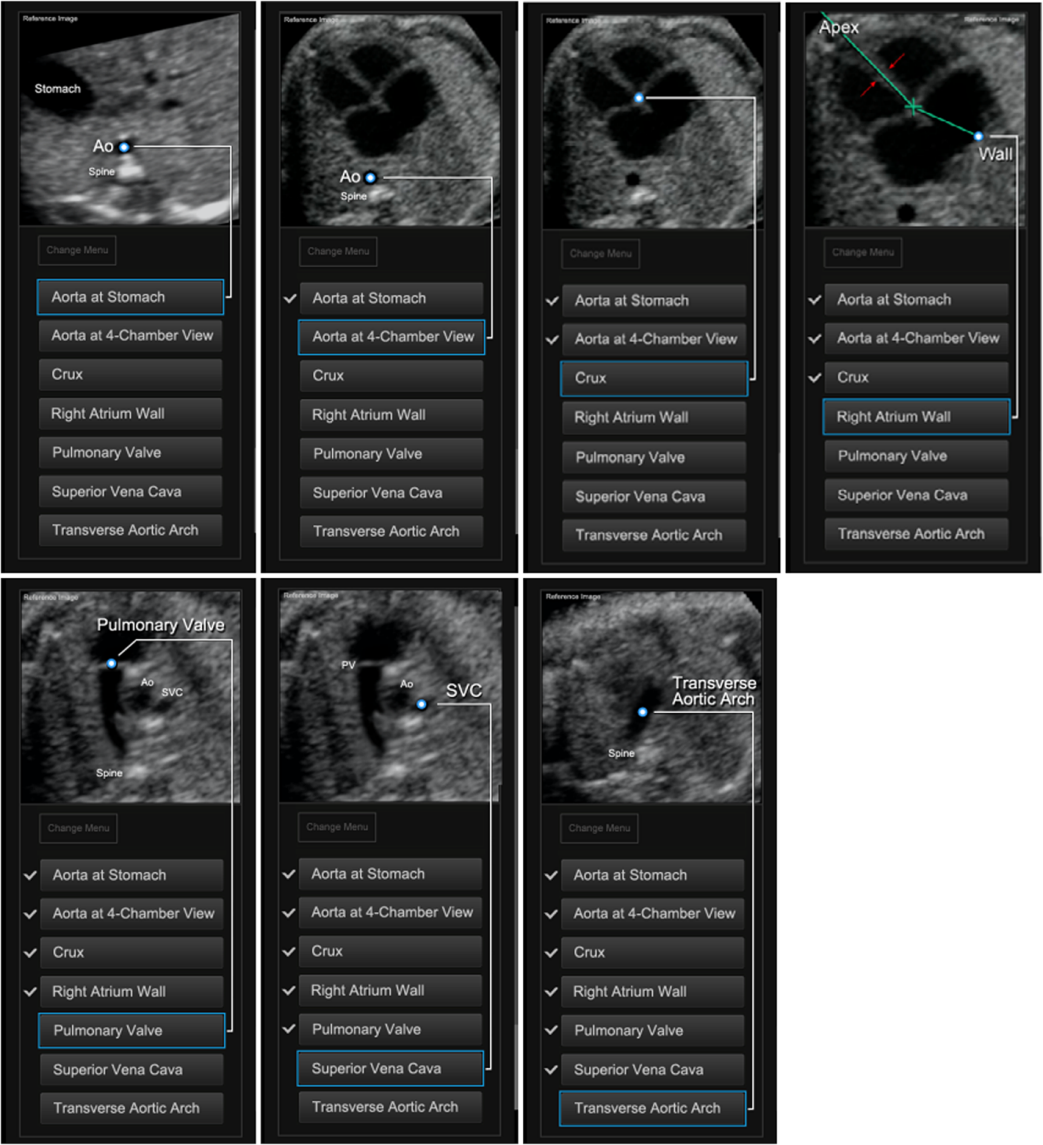
Seven anatomical structures within the heart that are marked using Anatomic Box®. The seven anatomical structures were as follows: 1) cross-section of the aorta at the level of the stomach; 2) cross-section of the aorta at the level of the four chamber view; 3) crux; 4) right atrial wall; 5) pulmonary valve; 6) cross-section of the superior vena cava; and 7) transverse aortic arch

**Fig. 2:**
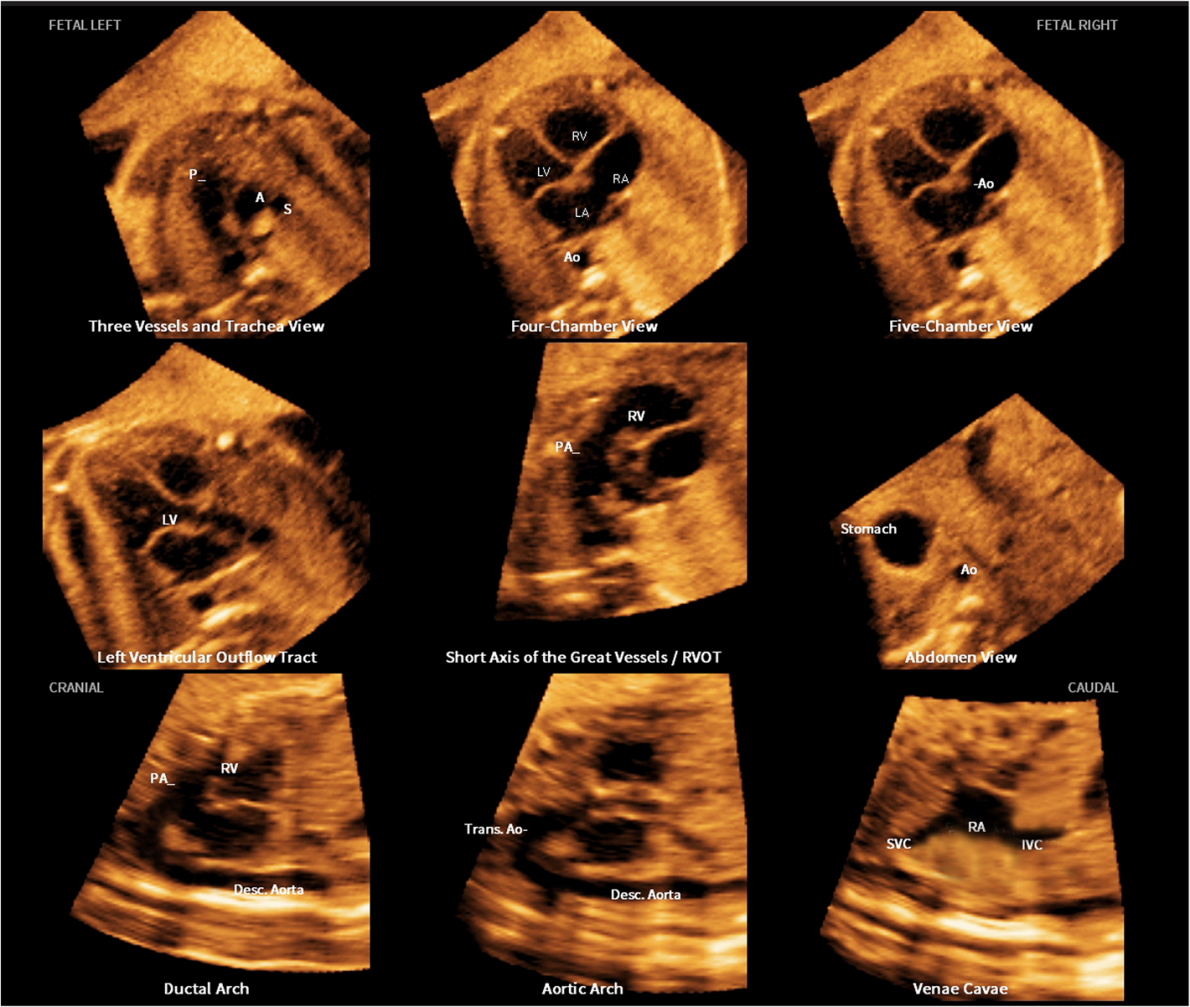
The nine diagnostic sections displayed by 5D Heart. The 9 diagnostic sections displayed by 5D Heart are as follows: 1) 3VT; 2) four chamber; 3) five chamber; 4) left ventricular outflow tract; 5) short-axis view of great vessels/right ventricular outflow tract; 6) abdomen/stomach; 7) ductal arch; 8) aortic arch; and 9) superior and inferior vena cava. STIC volume datasets of the foetal heart showing nine cardiac diagnostic planes with automatic labelling with 5D Heart in each plane, anatomical structures, foetal left and right sides and cranial and caudal ends

After marking seven anatomical structures of the foetal heart using the feature Anatomic Box® (Fig. 1), nine standard foetal echocardiography views were automatically generated and displayed with FINE (Fig. 2). The seven anatomical structures in sequential order are as follows: 1) cross-section of the aorta at the level of the stomach; 2) cross-section of the aorta at the level of the four chamber view; 3) crux; 4) right atrial wall; 5) pulmonary valve; 6) cross-section of the superior vena cava; and 7) transverse aortic arch (Fig. 1). Diagnostic planes were displayed approximately 3 s after marking.

### Elements and grading

With reference to the guidelines of the International Society of Ultrasound in Obstetrics and Gynecology, the 8 diagnostic sections and 29 diagnostic elements were studied [5, 8, 26]. Two less-experienced doctors (Observers A and B: J.H.Z. and X.Y.T.) and one experienced doctor (Observer C, B.W.Z.) scored the 29 elements from 8 basic diagnostic sections (we concluded that either the left ventricular outflow tract view or the five chamber view obtained was eligible in this study). All the Observers post-processed the images and scored them separately. The same volumes were reprocessed and scored by Observer A one month later to evaluated intra-observer agreement. When average scores of all diagnostic elements in each diagnostic section were more than or equal to 2 points, we deemed the section was successfully obtained in 5D Heart analysis.

**Table 1:** The success rates of 8 diagnostic planes with random four chamber view (*n* = 231)

Diagnostic planes	Displayed rate %(n)	95%CI
3VT	71.9%(166)	66.1–77.7%
Four chamber	76.2%(176)	70.7–81.7%
LVOT	83.1%(192)	78.3–87.9%
RVOT	61.9%(143)	55.6–68.2%
Abdomen/stomach	95.2%(220)	92.4–98.0%
Ductal arch	59.3%(137)	53.0–65.6%
Aortic arch	55.8%(129)	49.4–62.2%
SVC/RA/IVC	72.7%(168)	67.0–78.4%

*3VT* three-vessels and trachea view, *LVOT* left ventricular outflow tract view, *RVOT* right ventricular outflow tract view, *SVC/RA/IVC* superior and inferior vena cava/right atrium, *CI* confidence interval

**Fig. 3:**
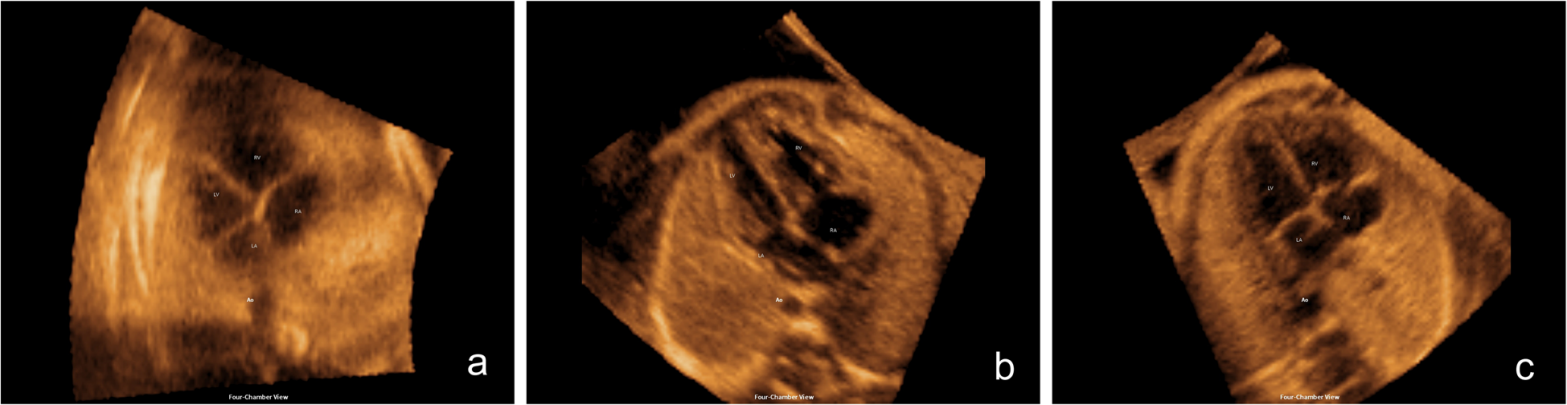
The three grades of image quality. A structure of interest that could be imaged clearly scored 3 points (**c**); adequately, 2 points (**b**); and unidentifiable, 1 point (**a**)

The diagnostic value was divided into 3 grades [27] according to the image quality (Fig. 3). A structure of interest that could be imaged clearly scored 3 points; adequately, 2 points; and unidentifiable, 1 point.

### Data analysis

We evaluated the success rate of each diagnostic plane obtained by the inexperienced physician (Observer A). The mean scores of each diagnostic element of 8 diagnostic sections were assessed, and the success rate of each diagnostic element was calculated. Inter- and intra-observer agreement on obtaining 8 diagnostic planes with 5D Heart were evaluated using Cohen’s kappa statistic. Values from 0.61 to 0.80 are considered substantial agreement; 0.81–1.00, near-perfect agreement. 95% confidence intervals (CI) of all statistics were calculated. All statistical analyses described above were performed with IBM SPSS package 21.0 (SPSS Inc., Chicago, IL, USA).

## Results

### The success rate of 5D Heart in the 8 diagnostic planes with foetal echocardiography

**Fig. 4:**
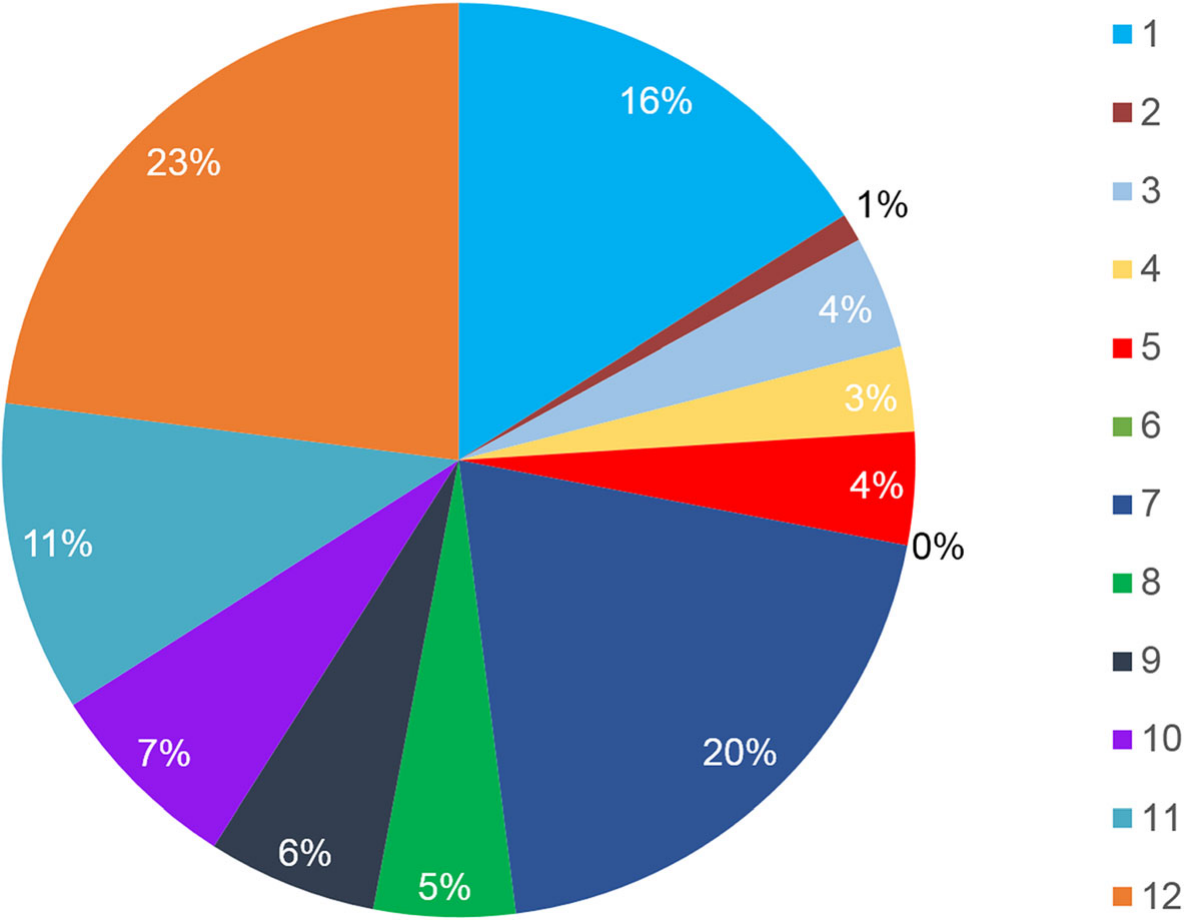
The components of foetal heart direction (n = 231). 1 to 12 means the apex of the foetal heart points between the 1- and 12- o’clock, and the rate of each direction is shown in the pie chart

**Fig. 5:**
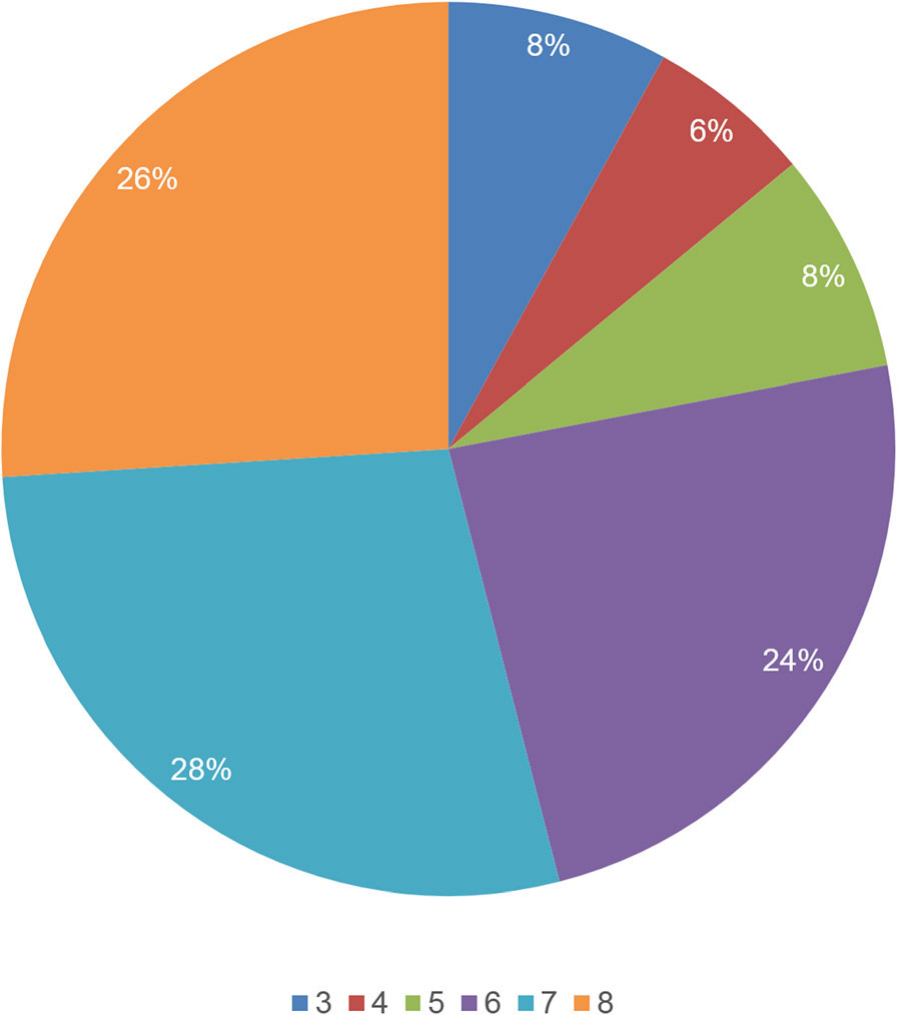
Number of foetal echocardiography views successfully obtained in each foetal heart with 5D Heart (*n* = 50). 3 to 8 means the total number of diagnostic planes retrieved from one foetus, and the success rates are shown in the pie chart

A total of 262 volume datasets were obtained in 209 normal foetuses, and 231 volume diagnostic sections were successfully obtained with the 5D Heart technique. Finally, 88.2% of the 262 volume datasets could be annotated. Observer A scored all diagnostic elements in each plane. Only when average scores of all diagnostic elements in each diagnostic section (such as LVOR, RVOT and so on) were more than or equal to 2 points was this plane considered to be successful in 5D Heart analysis. The results of 8 diagnostic sections were analysed, and the success rate of each diagnostic section is shown in Table 1. The operator who used 5D Heart for image post-processing was a less-experienced physician (Observer A). However, as shown in Table 1, we found that the success rates of 6 sections were more than 60% with the exception of the ductal arch view and aortic arch view with the random four chamber view as the initial section. Our study included all foetal heart positions, including when the apex of heart pointed between the 5- and 7- o’clock. The rate of each direction is shown in Fig. 4. Moreover, when the apical four chamber view was the initial section (apex pointed between the 12- and 1- o’clock), the success rate of each diagnostic section was higher, which is shown in Table 2. The success rate of each diagnostic view was up to 70.7–99.0%. Moreover, we randomly selected 50 foetuses to calculate the number of display sections in each foetal heart, and the statistics are shown in Fig. 5. In this study, all foetuses were obtained more than three diagnostic planes, 78% of foetuses were obtained more than six diagnostic planes successfully.

**Table 2:** The success rates of 8 diagnostic planes with apical four chamber view with 5D Heart (*n* = 99)

Diagnostic planes	Displayed rate %(n)	95%CI
3VT	81.8%(81)	74.2–89.4%
Four chamber	91.9%(91)	86.5–97.3%
LVOT	91.9%(91)	86.5–97.3%
RVOT	70.7%(70)	61.7–79.7%
Abdomen/stomach	99.0%(98)	95.3–100%
Ductal arch	74.7%(74)	66.1–82.3%
Aortic arch	70.7%(70)	61.7–79.7%
SVC/RA/IVC	74.7%(74)	66.1–83.3%

*3VT* three-vessels and trachea view, *LVOT* left ventricular outflow tract view, *RVOT* right ventricular outflow tract view, *SVC/RA/IVC* superior and inferior vena cava/right atrium, *CI* confidence interval

### The success rate of each diagnostic element in the 8 diagnostic sections obtained with 5D Heart

We further explored the success rate of each diagnostic element in the 8 diagnostic sections obtained with 5D Heart. The results are shown in Table 3. The success rate of each diagnostic element was 58.9%~ 100%.

**Table 3:** The display rates of elements of each diagnostic plane

Diagnostic planes	Diagnostic elements	Displayed rate%(n)	95%CI
3VT	pulmonary artery	95.7%(221)	93.1–98.3%
	aorta	94.8%(219)	91.9–97.7%
	superior vena cava	85.3%(197)	80.7–89.9%
	trachea	79.2%(183)	74.0–84.4%
Four chamber	four atrioventricular chambers	100%(231)	98.7–100%
	foramen ovale	99.6%(230)	97.5–100%
	crux	99.6%(230)	97.5–100%
	at least one pulmonary vein into the left atrium	83.1%(192)	78.3–87.9%
	interventricular septum	100%(231)	98.7–100%
	moderator bundle	91.8%(212)	88.3–95.3%
	atrioventricular valve	100%(231)	98.7–100%
	atrioventricular valve distance	99.6%(230)	97.5–100%
LVOT	aortic originated in left ventricle	91.8%(212)	88.3–95.3%
	interventricular septum	97.4%(225)	95.3–99.5%
	aortic valve	84.4%(195)	79.7–89.1%
RVOT	pulmonary artery originated in right ventricle	90.5%(209)	86.7–94.3%
	pulmonary bifurcation	65.4%(151)	59.3–71.5%
	pulmonary valve	84.4%(195)	79.7–89.1%
	aortic and pulmonary artery cross relationship	83.1%(192)	78.3–87.9%
Abdomen/stomach	stomach	96.5%(223)	94.1–98.9%
	abdominal aorta	97%(224)	94.8–99.2%
Ductal arch	arterial duct	65.8%(152)	59.7–71.9%
	arterial duct and pulmonary artery connection	73.2%(169)	67.5–78.9%
	ascending aorta	88.3%(204)	84.2–92.4%
Aortic arch	aortic arch	58.9%(136)	52.6–65.2%
	ascending aorta	74%(171)	68.3–79.7%
SVC/RA/IVC	superior vena cava	85.3%(197)	80.7–89.9%
	inferior vena cava	80.1%(185)	75.0–85.2%
	right atrium	94.4%(218)	91.4–97.4%

*3VT* three-vessels and trachea view; LVOT: left ventricular outflow tract view, *RVOT* right ventricular outflow tract view, *SVC/RA/IVC* superior and inferior vena cava/right atrium, *CI* confidence interval

### The assessment of 5D Heart operator dependency

Observer A and Observer B were both less-experienced doctors but had been engaged in ultrasound diagnosis for more than 5 years and had at least 6 months of systematic training for foetal echocardiography. Observer C was an expert in foetal echocardiography. The agreement of Observer A and Observer B with Observer C was assessed by Cohen’s kappa. Values of kappa ranged from 0.769 to 1.000, indicating substantial agreement to near-perfect agreement. The data are presented in Table 4.

**Table 4:** Inter-observer agreement between two less experienced doctor (Observer A and Observer B) and experienced doctor (Observer C) with 5D Heart in obtaining 8 diagnostic planes

Diagnostic Planes	Observer A with Observer C		Observer B with Observer C	
	kappa-value	95%CI	kappa-value	95%CI
3VT	0.955	0.892–1.000	0.932	0.856–1.000
Four chamber	1.000	0.987–1.000	1.000	0.987–1.000
LVOT	0.958	0.899–1.000	0.959	0.902–1.000
RVOT	0.801	0.705–0.897	0.822	0.728–0.916
Abdomen/stomach	0.769	0.551–0.987	1.000	0.987–1.000
Ductal arch	0.970	0.937–1.000	0.960	0.921–0.999
Aortic arch	0.878	0.815–0.941	0.815	0.741–0.889
SVC/RA/IVC	0.881	0.805–0.957	0.813	0.715–0.911

*3VT* three-vessels and trachea view, *LVOT* left ventricular outflow tract view, *RVOT* right ventricular outflow tract view, *SVC/RA/IVC* superior and inferior vena cava/ right atrium, *CI* confidence interval

### The estimate of 5D Heart reproducibility

Inter-observer reliability was evaluated by agreement of Observer A and Observer B. The inter-observer reliability was high and the results are shown in Table 5. Intra-observer reliability was evaluated by agreement of Observer A in obtaining 8 diagnostic planes at different time points (the time interval was one month), and substantial to near-perfect agreement was found. The results are shown in Table 6.

**Table 5:** Inter-observer agreement between two less experienced doctors in obtaining 8 diagnostic planes

Diagnostic Planes	kappa-value	95%CI
3VT	0.932	0.856–1.000
Four chamber	1.000	0.987–1.000
LVOT	0.938	0.869–1.000
RVOT	0.612	0.487–0.737
Abdomen/stomach	0.769	0.551–0.987
Ductal arch	0.930	0.879–0.981
Aortic arch	0.693	0.599–0.787
SVC/RA/IVC	0.652	0.523–0.779

*3VT* three-vessels and trachea view; LVOT: left ventricular outflow tract view, *RVOT* right ventricular outflow tract view, *SVC/RA/IVC* superior and inferior vena cava/ right atrium, *CI* confidence interval

**Table 6:** Intra-observer agreement of 5D Heart in obtaining 8 diagnostic planes

Diagnostic Planes	kappa-value	95%CI
3VT	0.770	0.627–0.913
Four chamber	1.000	0.987–1.000
LVOT	0.655	0.485–0.826
RVOT	0.656	0.517–0.795
Abdomen/stomach	1.000	0.987–1.000
Ductal arch	0.755	0.663–0.847
Aortic arch	0.615	0.517–0.713
SVC/RA/IVC	0.689	0.568–0.811

*3VT* three-vessels and trachea view; LVOT: left ventricular outflow tract view, *RVOT* right ventricular outflow tract view, *SVC/RA/IVC* superior and inferior vena cava/ right atrium, *CI* confidence interval

## Discussion

The prenatal diagnosis of CHDs remains a challenge. The difficulties of prenatal diagnosis are generally attributed to the complex anatomy of the heart as well as its motion and small size [8, 26]. Adequate examination of the foetal heart depends on foetal position and is time consuming, and expertise and skill are required [2, 10, 28]. It is particularly difficult for young sonographers to acquire 8 standard screening sections, especially with an aortic arch view and a ductal arch view [9, 26]. STIC technology allows the acquisition of a volume dataset of the foetal heart, in which cardiac planes may be extracted and displayed in any orientation [4, 12, 15]. However, extracting and displaying the recommended diagnostic planes from a volume dataset that can be dissected in many ways (i.e., planes) requires in-depth knowledge of anatomy and is difficult and operator-dependent [21, 23]. Planes and cardiac structures may be difficult to recognize, particularly when the anatomy is abnormal. Preliminary studies have shown that FINE generates and displays 8 standard foetal echocardiography views automatically after marking seven anatomical structures of the foetal heart (Fig. 3) [21, 22]. It allows the interrogation of volume datasets and the simultaneous display of up to three independent (non-orthogonal) planes by manually drawing straight or curved lines from any direction or angle. Garcia et al. [21] demonstrated that STIC volumes may be obtained successfully in approximately 75% of pregnancies with normal foetal hearts. By applying the FINE method to such volumes, 8 standard foetal echocardiography views can be generated in 98–100% of patients. However, all the published data acquired STIC volume datasets of the apical four chamber view only when the foetal spine was located between the 5- and 7-o’clock positions; this finding was not consistent with the findings of routine scans because, in some cases, apical four chamber views could not be obtained due to the different presentations. To the best of our knowledge, no data have been published previously with regard to its value for obtaining 29 key diagnostic elements in 8 standard sections in any foetal heart position.

To further confirm 5D Heart’s clinical value in routine screening, our study investigated 29 diagnostic elements obtained by 5D Heart in 8 standard foetal echocardiography views and the display rate of each section with random foetal heart position according to the guidelines of the International Society of Ultrasound in Obstetrics and Gynecology [1, 26]. An apex pointing to any direction from 1- to 12-o’clock was included in the present study, and the percentage of each position is shown in Fig. 4. Only when average of all diagnostic elements were more than 2 points (Fig. 3) was the section considered to be successful. The success rates of 8 standard planes with random foetal heart position were showed in Table 1. The data were lower with this view than with the apical four chamber view as the initial view (Table 2) but were more consistent with routine ultrasonic screening and with more important clinical value. The difference is considered to be partly due to the acoustic shadow of the spine and ribs. With obtaining 3D volume datasets by a volume probe, we found that when the scanning section started with the apical four chamber view (the apex pointed between the 9- and 3-o’clock), especially when the apex pointed between the 11- and 1-o’clock, this position could avoid the acoustic shadow of the spine and ribs compared with the basal apical four chamber view as the initial section. The excellent initial section inevitably led to better image quality. Our study included all four chamber views as the initial section, which is similar to daily work. Although the display rates of the planes were reduced, the success rates of the left ventricular outflow tract (LVOT), right ventricular outflow tract (RVOT), abdomen/stomach and superior and inferior vena cava long axis view were still 61.9%~ 95.2%. The above 6 sections showed a clear diagnosis of CHDs in most foetuses.

Additionally, the number of foetal echocardiography views successfully obtained through 5D Heart was calculated in 50 randomly selected data volumes (Fig. 5). For the diagnostic planes, 78% (*n* = 27) of STIC volumes demonstrated more than six echocardiography views. Recent studies have demonstrated that four chamber views can detect 30% of CHDs [8]. When the evaluation of outflow tracts was added to the four chamber view, the sensitivity of ultrasound screening for CHDs increased from approximately 30 to 69%–83% [29, 30]. Currently, the three vessel (3 V) and 3 V with trachea (3VT) views were added to the standard four chamber and outflow views to improve the detection of CHDs [31–33]. The latter enabled the detection of lesions such as coarctation of the aorta, right aortic arch, double aortic arch, and vascular rings, achieving a prenatal detection rate of congenital heart disease up to 90% [29]. 5D Heart can obtain more than three planes successfully in all foetuses and can acquire more than five planes in 78% of foetal hearts. We therefore speculated that 5D Heart could be a simple method to visualize the foetal heart. The relatively lower detection rate of the four chamber view in the random four chamber view than the success rate of 5D Heart for obtaining 8 standard diagnostic sections (the volumes were acquired with the four chamber view) shows that there are several factors that may contribute to the results. First, some volumes’ image quality were not good enough because they could be affected by breath of pregnant woman and foetal movement. Second, because average of all diagnostic elements in the diagnostic view were more than or equal to 2 points, we deemed this diagnostic view successful for retrieval in 5D Heart analysis. Third, volumes acquired with the random four chamber view as the initial section including the apex located between the 5- and 7-o’clock, image quality was degraded in this position due to the shadows of the ribs and spine.

To further study the value of 5D Heart in diagnostic components, the success rates of 29 diagnostic elements were semi-quantitatively scored [27] in 8 standard foetal echocardiography views (Table 3). We considered only successful sections for analysis of the main structure of the foetal cardiovascular system. The success rates of the diagnostic elements ranged from 73.2 to 100% with the exception of the pulmonary bifurcation (65.4%), arterial duct (65.8%) and aortic arch (58.9%). Among them, 15 elements’ success rates were more than 90%, demonstrating that image quality displayed by 5D Heart was excellent. Moreover, by using 5D Heart, the diagnostic time was shortened for the foetal heart. The acquired volumes ranged from 7 to 10 s. Marking seven anatomical structures was finished within 1 min, and diagnostic planes were displayed 3 s after marking. In total, the 8 standard diagnostic sections speculated were displayed within 10 min by 5D Heart. However, routine echocardiography screening requires more than 15 min for experts and over 30 min for less-experienced physicians [9, 10]. In addition, a large number of foetuses could not be displayed in all 8 standard sections, especially the aortic arch view and ductal arch view, by less-experienced physicians. Therefore, the use of 5D Heart increases efficiency in routine screening, and one physician can complete more cases in the same time as with 2D ultrasound. This technology could probably be useful for alleviating the burdens on sonologists in developing countries. However, the actual efficiency of this method requires further investigation.

Previous studies reported low sensitivity (22.5–52.8%) for the detection of CHDs [5, 21, 26]. This outcome has been attributed mainly to operator expertise and experience [6, 11]. Experienced sonographers perform significantly better than less-experienced sonographers in terms of obtaining diagnostic views and detecting CHDs [1, 6, 11]. However, our study showed substantial to near-perfect agreement between less-experienced sonographers and experts, indicating that young operators performed as well as experts in terms of the use of 5D Heart in the foetal heart. The result was in agreement with Paladini et al. [19]. Experienced sonographers perform significantly better in terms of assessing the ascending aorta and pulmonary artery in screening examinations. However, no difference has been found for obtaining four chamber views [7, 9]. Volume datasets were acquired in the four chamber view with 5D Heart technology in the present study. Inter-observer agreement was excellent between less experienced sonographers and experienced sonographers, we speculated that 5D Heart was not operator-dependent in the foetal heart. Therefore, 5D Heart may be a novel and automatic method for visualizing eight standard foetal echocardiography views with low operator dependence. Less-experienced sonographers, including screening sonographers and foetal echocardiographers, would benefit from this technology. 5D Heart is expected to be a new approach to promote foetal heart exams worldwide, especially in communities or remote regions, by less-experienced physicians. After a short training period (approximately one week), doctors in district and community hospitals can perform foetal heart examinations with 5D Heart. When the foetal heart is abnormal or suspected to be abnormal due to their knowledge, physicians can complete the foetal heart scan with 5D Heart and store the volume datasets; then, they can transmit these datasets to experienced physicians in superior hospitals for remote consultation. This approach is expected to reduce unnecessary travel for pregnant women and reduce unnecessary risks during pregnancy. Moreover, these volume datasets can be stored for future reference and studies.

The inter-observer and intra-observer variability of 5D Heart was assessed by agreement of two physicians with relatively poor experience and the agreement of the same physician at different times (the time interval was one month). The kappa values ranged from 0.612 to 1.000, suggesting that 5D Heart has good reproducibility. The results are shown in Tables 5 and 6.

However, 5D Heart has some disadvantages. First, it has relatively poorer 3D imaging spatial and temporal resolution compared to 2D imaging; additionally, it lacks interactions for avoiding acoustic shadowing during a fixed volume acquisition compared to a real-time 2D scan; and finally, the imaging quality will be degraded by motion artefacts, even with the use of STIC.

In addition, this study has several limitations. First, all volume datasets in this study were acquired by an expert, and differences between physicians in terms of obtaining datasets need further study. In addition, in the volume dataset acquisition, the factors that affect the quality of 2D echocardiography images can also affect 5D Heart, such as the shadowing of the spine and ribs, foetal movement and so on. Second, we did not evaluate the cardiac rate, rhythm disturbances or Doppler velocimetry of foetal heart in this study. Additionally, this study also does not include any abnormalities. Therefore, the ability of this method to be used generally merits further investigation. In addition, this study provides information limited to 20–40 weeks of gestation. Further studies need to include foetuses with a lower gestation age.

## Conclusions

In summary, our study is the first to demonstrate that 5D Heart based on FINE can easily display the main diagnostic factors in 8 basic foetal echocardiography sections. 5D Heart improves diagnostic elements with ease of operation, efficiency, examiner independency and good repeatability. Furthermore, once the datasets are obtained and stored, they are convenient and useful for teaching, remote consultation and research. However, applying this technology clinical use requires further study and more practice. In conclusion, 5D Heart is a feasible, accurate and operator-independent method for foetal heart examination during the second and third trimesters.

## Data Availability

The datasets used and analyzed in the study are available from the corresponding author on reasonable request.

## Abbreviations

2D: Two-dimensional
3 VT: Three trachea view
3D: Three-dimensional
3V: Three vessel view
4D: Four-dimensional
CHDs: Congenital heart diseases
CI: Confidence interval
FINE: Fetal intelligent navigation echocardiography
LVOT: Left ventricular outflow tract
RVOT: Right ventricular outflow tract
STIC: Spatiotemporal image correlation
